# Femtosecond Laser-Ablated Copper Surface as a Substrate for a MoS_2_-Based Hydrogen Evolution Reaction Electrocatalyst

**DOI:** 10.3390/ma15113926

**Published:** 2022-05-31

**Authors:** Ramūnas Levinas, Asta Grigucevičienė, Tadas Kubilius, Aidas Matijošius, Loreta Tamašauskaitė-Tamašiūnaitė, Henrikas Cesiulis, Eugenijus Norkus

**Affiliations:** 1 State Research Institute Center for Physical Sciences and Technology (FTMC), Saulėtekio Ave. 3, LT-10257 Vilnius, Lithuania; asta.griguceviciene@ftmc.lt (A.G.); loreta.tamasauskaite@ftmc.lt (L.T.-T.); 2 Faculty of Chemistry and Geosciences, Vilnius University, Naugarduko Str. 24, LT-03225 Vilnius, Lithuania; henrikas.cesiulis@chf.vu.lt; 3 Hydrogen Solutions Ltd., Partizanų Str. 61-806, LT-49282 Kaunas, Lithuania; tk@h2solutions.lt; 4 Laser Research Center, Vilnius University, Saulėtekio Ave. 10, LT-10223 Vilnius, Lithuania; aidas.matijosius@ff.vu.lt

**Keywords:** femtosecond laser, hydrogen evolution, electrocatalysis, molybdenum sulfide, scanning electrochemical microscopy, electrochemical impedance spectroscopy

## Abstract

One of the methods to improve the performance of a heterogeneous electrocatalyst is the dispersion of a catalytic material on a suitable substrate. In this study, femtosecond laser ablation was used to prepare very rough but also ordered copper surfaces consisting of vertical, parallel ridges. Then, a molybdenum sulfide coating was electrochemically deposited onto these surfaces. It was observed by profilometry that the average roughness of the surface after coating with MoS_2_ had decreased, but the developed surface area still remained significantly larger than the projected surface area. The electrodes were then used as an electrocatalyst for the hydrogen evolution reaction in acidic media. These were highly efficient, reaching 10 mA cm^−2^ of HER current at a −181 mV overpotential and a Tafel slope of ~39 mV dec^−1^. Additionally, scanning electrochemical microscopy was used to observe whether hydrogen evolution would preferentially occur in certain spots, for example, on the peaks, but the obtained results suggest that the entire surface is active. Finally, the electrochemical impedance spectroscopy data showed the difference in the double-layer capacitance between the ablated and non-ablated surfaces (up to five times larger) as well as the parameters that describe the improved catalytic activity of fs-Cu/MoS_2_ electrodes.

## 1. Introduction

The use of femtosecond (fs) lasers through a process called laser ablation in material processing has grown substantially in the last two decades [1,2,3,4] and is motivated by unparalleled control of the processing parameters enabled by such a light source [5]. Among the other fields, surface structuring has benefited greatly from the usage of fs lasers [6]. Here, it allows one to achieve true hierarchical surface features on a large variety of substrates such as various metals [7], glasses [8], or polymers [9]. Therefore, it comes as no surprise that surfaces made using a fs light source have been tested in a vast array of applications including self-cleaning [10], anti-icing [11], anti-fouling [12], photonics, and bionics [13,14], to name a few. Moreover, by optimizing the processing parameters, new phases can be formed and modified on the surface by a process called laser alloying [15].

On the other hand, electrochemical catalysis is constantly searching for nano- or micro-structured substrates with a significant volume-to-surface-area ratio such as metallic foams [16]. There have already been reports of the strongly enhanced hydrogen evolution reaction (HER) electrocatalytic activity of fs-blackened Cu compared to an unmodified sample [17]. Moreover, several studies on fs-treated Ni have revealed its enhanced oxygen evolution reaction (OER) [18,19] and HER [20] electrocatalytic properties in alkaline media. A broad range of transition metals including Ti, Mo, W, Co, and more have been investigated for their enhanced electrocatalytic HER activity after laser ablation [21]. In these cases, improved performance was related to a larger specific surface area created by fs-treatment.

The beneficial effect of femtosecond laser treatment on metallic surfaces for their electrocatalytic properties is certain, but there is also the possibility of combining these evolved morphologies with other catalytic materials. A potentially interesting method for electrocatalyst synthesis has been reported recently, where fs-laser treatment was applied on nickel surfaces immersed in aqueous salt solutions to form Ni-Mo-Fe alloys [22]. Another way to combine fs-treatment with electrocatalysis is to coat an already treated substrate with an electrocatalytic material. In a recent study, a Co oxide/CuO/Cu electrocatalyst with enhanced OER/HER catalytic properties was constructed from fs-treated Cu and a hydrothermally synthesized cobalt oxide coating [23].

The conductive nature of laser-ablated metal surfaces lends itself to the application of electrochemical deposition. For electrocatalytic applications, many different catalytic materials can be electrodeposited on various conductive surfaces [24,25,26]. The factor that determines the catalyst selection is its desired properties such as electrocatalytic activity in certain media (acidic or alkaline), efficiency, scalability, and even cost. The latter is why transition metal chalcogenides have received much attention in the field of electrochemical water splitting, as they are relatively inexpensive when compared to precious-metal-based (e.g., platinum) catalysts. In particular, molybdenum disulfide (MoS_2_) is interesting because it is a non-precious metal catalyst with an electrocatalytic activity that can approach that of platinum for HER [27,28].

MoS_2_ coatings and films of various morphologies and structures can be prepared by various methods: wet-chemical [29] or hydrothermal synthesis [27,30], ultrasonic spray pyrolysis [31], electrochemical deposition [24,28,32,33], and more. Electrodeposition, in particular, is interesting, because it allows a conductive electrode surface to be covered homogeneously with a semiconducting but electrocatalytic MoS_2_ film. Additionally, the process is relatively simple, can be carried out at room temperature, and typically yields amorphous and highly electrocatalytically active films. The properties of the electrochemically synthesized films can be tuned by adding certain surfactants/reducing agents such as sodium dodecyl sulfate [28] or sodium hypophosphite [34] into the electrodeposition solution. The former was found to have a beneficial effect on the HER electrocatalytic stability of the MoS_2_ film, and the latter was observed by X-ray photoelectron spectroscopy (XPS) to promote the formation of Mo(IV)-S bonds and slightly improve the catalytic efficiency of the film.

Therefore, the aim of this study was to combine the surface engineering capabilities of femtosecond laser ablation with the electrocatalysis of the hydrogen evolution reaction by electrochemically deposited MoS_2_. To this end, copper surfaces were subjected to “femtosecond blackening” (i.e., femtosecond laser ablation) to prepare unidirectional ridged mesostructures. Then, the surfaces with evolved morphologies were coated with electrocatalytic MoS_2_ by electrochemical deposition. The structures and surface morphologies of the uncoated copper surfaces and catalytic electrodes were extensively characterized. The prepared electrodes were tested for their HER electrocatalytic activity in acidic media, paying attention to both the total electrode activity as well as the local electrocatalytic activity observations by scanning electrochemical microscopy.

## 2. Materials and Methods

### 2.1. Femtosecond Blackening

A general femtosecond direct laser write system such as the one as in [35] was used. It comprised a femtosecond laser with a central wavelength of 1030 nm, pulse duration of 750 fs, a repetition rate of 500 kHz, and a galvanometric scanner with an F-theta lens (focal distance 100 mm). The maximum working field of the optic was 45 × 45 mm. Treated surfaces were designated as fs-Cu.

### 2.2. MoS_2_ Electrodeposition

The MoS_2_ films were synthesized by electrochemical cathodic deposition from a tetrathiomolybdate (MoS_4_^2−^/TTM) solution that had been synthesized by the chemical sulfidation of (NH_4_)_6_Mo_7_O_24_ (≥99%, Roth) with Na_2_S xH_2_O (60%, Roth). The composition of the solution was as follows: 0.025 M MoS_4_^2−^, 0.1 M Na_2_SO_4_ (≥99%, Roth), 0.1 M NaH_2_PO_2_ (≥99%, Roth). The pH of the electrolyte was ~8. Sodium hypophosphite was added because it was found to have a beneficial effect on the Mo-S bond formation during cathodic MoS_2_ deposition in a previous study [34]. The depositions were carried out under potentiostatic conditions at −1.1 V vs. Ag/AgCl by limiting the amount of charge passed through the system to 5 C. The cathodic deposition of MoS_2_ occurs as in Equation (1) [36].

MoS_4_^2−^ + 2e^−^ + 2H_2_O → MoS_2_ + 2SH^−^ + 2OH^−^ (1)

The MoS_2_ films were deposited on plain Cu (Cu/MoS_2_) and fs-blackened Cu (fs-Cu/MoS_2_) substrates. The plain Cu was abraded with 1200 grit sandpaper in circular motions until the surface visually appeared to be homogeneously rough. This is necessary because when deposited on a mirror-smooth copper substrate for 5 C, the MoS_2_ films would readily delaminate with hydrogen bubbling.

The surface morphology and elemental composition of the electrodeposited MoS_2_ films were evaluated using a scanning electron microscope (SEM) TM 4000 plus with an AZetecOne detector (Hitachi Ltd., Tokyo, Japan) for the energy-dispersive X-ray spectroscopy (EDX) measurements. Energy-dispersive X-ray spectroscopy was used to determine the elemental and phase composition of the electrodes and electrodeposited catalysts. An X-ray diffractometer (D2 Phaser, Bruker, Billerica, Massachusetts, USA, λ = 1.5418 Å/Cu Kα) was used.

### 2.3. Characterization of HER Electrocatalytic Activity

All electrochemical experiments (potentiostatic electrodeposition, linear sweep voltammetry (LSV), electrochemical impedance spectroscopy (EIS)) were performed in a standard three-electrode cell using a potentiostat/galvanostat Autolab 302N (Metrohm, Utrecht, The Netherlands). Unless specified otherwise, all potentials are given as overpotentials (η) in reference to the reversible hydrogen electrode (RHE). Electrocatalytic activity characterization was carried out in 0.5 M H_2_SO_4_ by the following procedure: (1) settling of open circuit potential (OCP) for 120 s; (2) 3 LSV sweeps at a rate of 2 mV s^−1^ from 0 V to a cut off condition of −100 mA cm^−2^ as normalized by geometric surface area; and (3) EIS measurements at −0.1 V, −0.2 V, −0.3 V overpotential. The EIS spectra were obtained in the frequency range from 10 kHz to 100 mHz, with a perturbation amplitude of ±10 mV. The electrochemical impedance data were modeled with equivalent electric circuits (EEC) using Zview software. The determined solution resistance R_s_ was used to correct the ohmic drop for Tafel slope analysis.

### 2.4. Profilometrical Analysis

The surface roughness and morphology of the samples with and without modification were evaluated by means of a 3D optical profiler ContourGT-K (Bruker Nano GmBH, Berlin, Germany) in non-contact mode using white light and phase shift interferometry. For each measurement, 50× optics was used to scan the surface area of 500 μm × 500 μm. Then, the parameters of the surface characteristics such as surface roughness (S_a_—arithmetic mean of the absolute departures of the roughness profile from the mean line), maximum peak height above the mean line (S_p_), maximum valley depth below the mean line (S_v_), and the projected and developed surface areas (S_par_, S_dar_) were evaluated. Vision64 and Profilm Online software was used for data acquisition and surface analyses.

### 2.5. Scanning Electrochemical Microscopy (SECM)

A Versa SCAN (Ametek, Berwyn, PA, USA) SECM workstation was used in connection with a Versa STAT 3 and Versa STAT 3F bipotentiostat (Princeton Applied Research, Oak Ridge, TN, USA). The substrate-generation tip-collection (SG/TC) mode was used, where the sample being investigated was used to generate hydrogen, and the tip would oxidize the generated products. The electrolyte was 0.5 M H_2_SO_4_, as previously used. The probe was a Pt ultramicroelectrode with a diameter of 10 μm. The probe was positioned ~10 μm above the sample and a constant height mode was used, which may be prone to errors from uneven sample mounting. However, possible errors were minimized by examining a small area (X–Y 100 × 150 μm) and a 5 μm s^−1^ tip scan rate. The SECM maps presented in the discussion are 100 × 100 μm because the first 100 × 50 μm data were typically distorted by the settling of either the substrate generation or tip collection current. The first scan lines were considered as pretreatment and discarded.

## 3. Results and Discussion

### 3.1. Profilometrical Analysis, Surface Morphology and Structure

**Figure 1:**
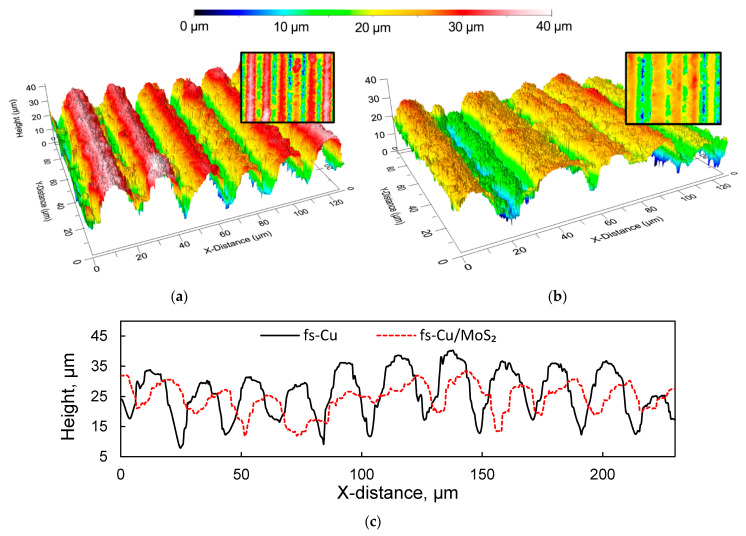
The 3D profilograms of the (**a**) fs-Cu surface; (**b**) a fs-Cu/MoS_2_ surface; (**c**) 2D slices across the *Y* axis.

Optical profilometry was employed to obtain the initial information on the morphology of the fs-blackened copper surfaces. The profilogram of the fs-Cu sample showed that femtosecond processing created a homogeneous structure comprised from ordered rows of peaks and pits (Figure 1a). The peaks had a width of up to 20 µm and the pits were typically narrower than 10 µm. As can be seen from a 2D slice across the *Y* axis, the peak-to-pit height difference was 10 to 25 µm (Figure 1c). The area roughness analysis data are shown in Table 1. The mean height S_a_ = 6.86 µm (as measured across the 0.5 × 0.5 mm profilogram shown in Figure S1), valley depth S_v_ was 53.11 µm, and the peak height S_p_ was 24.26 µm. The projected surface area (S_par_) was 0.25 mm^2^, whereas the developed surface area (S_dar_) was 10.46 mm^2^, signifying the enhanced roughness. An identical analysis was performed on the fs-Cu/MoS_2_ sample in order to estimate whether the electrochemically deposited film had changed the surface topography. There appeared to be a narrow peak in this profilogram (Figure 1b), but this is most likely related to the femtosecond ablation process and is not the effect of the electrochemical MoS_2_ deposition. Overall, the surface became less rough, the S_a_ decreased to 4.94 µm, the S_v_ to 48.36 µm, and S_p_ to 16.21 µm. Consequently, S_dar_ = 4.68 mm^2^, which was under half of what had been approximated for fs-Cu. The smoother surface was also reflected in the 2D slice, where it is evident that the peak-to-pit height ratio had decreased. These results are expected because a significant amount of the catalytic material was deposited. However, the decrease in roughness may signal that more MoS_2_ is deposited in pits rather than on peaks, thus leveling out the surface. This analysis already implies that assuming a known geometrical surface area of an electrocatalytic electrode is at best inaccurate; this issue will later be addressed with non-stationary electrochemical methods.

**Figure 2:**
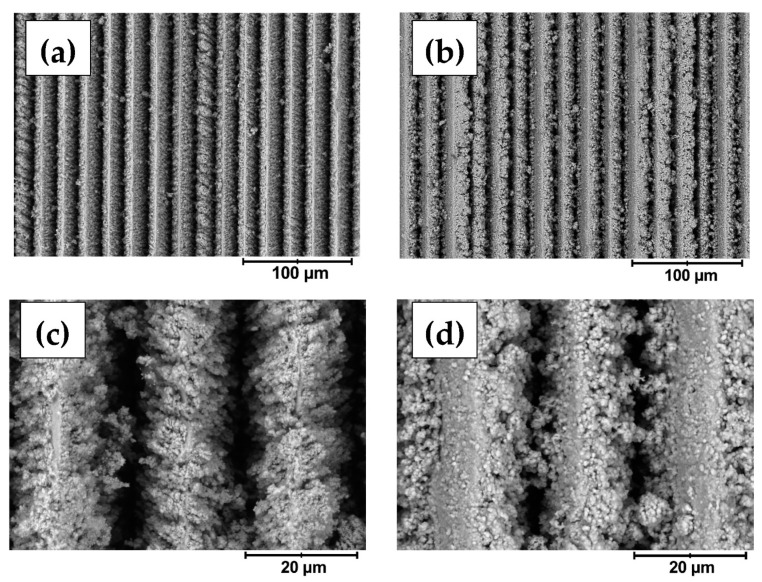
The SEM images of the (**a**,**c**) fs-Cu, and (**b**,**d**) fs-Cu/MoS_2_ surfaces.

The surface morphology was observed in detail by SEM. In agreement with the profilometry, the fs-Cu surfaces consisted of ordered parallel ridges, and the width of a single ridge or pit-to-pit distance was ~20 µm (Figure 2a). At higher magnifications, it became apparent that femtosecond laser ablation in fact created a very coarse microstructure and that the edges of the peaks were rough (Figure 2c). When a MoS_2_ film is electrochemically deposited on the fs-Cu substrate, several things can be noted. The MoS_2_ deposits in both the peaks and pits, but it seems that more material is deposited within the pits (Figure 2b,d). Especially from higher magnification images, it can be seen that MoS_2_ seems to preferentially crystallize on the edges of the peaks, occasionally even forming bridges between them. In contrast, the morphology of the deposited MoS_2_ on the tops of the peaks was rather flat. This is likely to be the cause of the decrease in the surface roughness that was previously observed by profilometry—more material is simply deposited within the pits of the fs-ablated copper surface. Such deposition kinetics may have been caused by the higher local current densities that are formed within the pits, thus causing faster MoS_2_ electrochemical deposition.

Regarding the atomic composition, the EDX measurements showed an average S to Mo atomic % ratio of 2.8 (i.e., a stoichiometry of MoS_2.8_). MoS_3_ is known to exist but is typically formed by the chemical disproportionation of [MoS_4_]^2−^ at anodic potentials [37]. It is likely that here, unreacted S^2−^ or HS^−^ in the solution could have interacted with the copper substrate, thus increasing the overall content of sulfur in the atomic composition of these films. The XRD analysis revealed that, in accordance with other research on electrochemically deposited MoS_2_, the films were amorphous (Figure S2) [28,38]. No peaks that could be assigned to any phase of MoS_2_ were seen. Because the films were relatively thin, there remains a strong signal from the substrate. It is worth noting that the (200) peak became the most intense after depositing a MoS_2_ film on the fs-Cu, which probably shows that MoS_2_ tends to deposit on the (111) plane. Moreover, an XPS analysis carried out on similarly synthesized films in a previous study confirmed the formation of Mo(IV)-S bonds [34].

### 3.2. Electrocatalytic Activity

**Figure 3:**
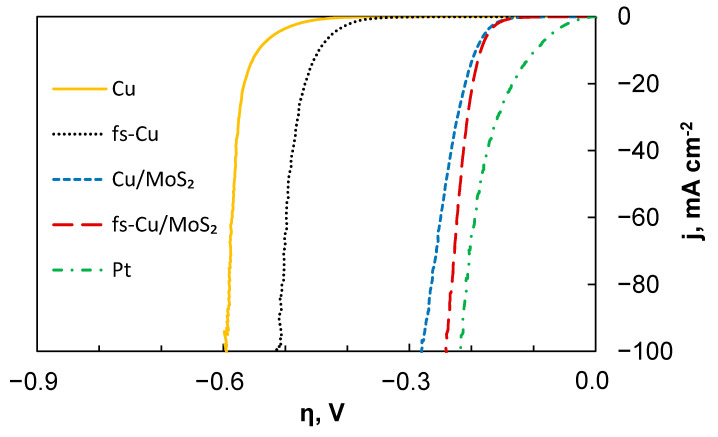
The LSV curves showing the HER electrocatalytic activity of the unmodified and modified copper substrates as well as the respective substrates coated in an electrochemically deposited MoS_2_ film. Scan rate—2 mV s^−1^, 0.5 M H_2_SO_4_ electrolyte. Corrected for iR-drop.

The HER electrocatalytic activity of the samples was initially investigated by linear sweep voltammetry in 0.5 M H_2_SO_4_. In each experiment, three curves were obtained, and Figure 3 shows the third curve for each sample. Here, iR correction was applied by using the solution resistance value that was obtained from the high frequency part of the EIS spectra, which will be discussed later. It must be noted that the current density was normalized to the geometric surface area of the substrate and the experiment represented the total electrode activity, which is heavily influenced by the actual electrochemically active surface area. The plain Cu exhibited the poorest electrocatalytic activity, whereas the fs-Cu without any catalyst coating already showed a marginally better performance. Again, it is important to note that this is most likely related to the increased electrochemically active surface area. When the MoS_2_ films form on these surfaces, the catalytic activity increases significantly (i.e., higher current densities are reached at lower overpotentials). The electrode that was prepared by electrochemically depositing the MoS_2_ onto the copper foil treated with abrasive paper (Cu/MoS_2_) showed good electrocatalytic performance. However, the highest catalytic activity (apart from platinum, which is presented for comparison) was achieved with the fs-Cu/MoS_2_ sample.

**Figure 4:**
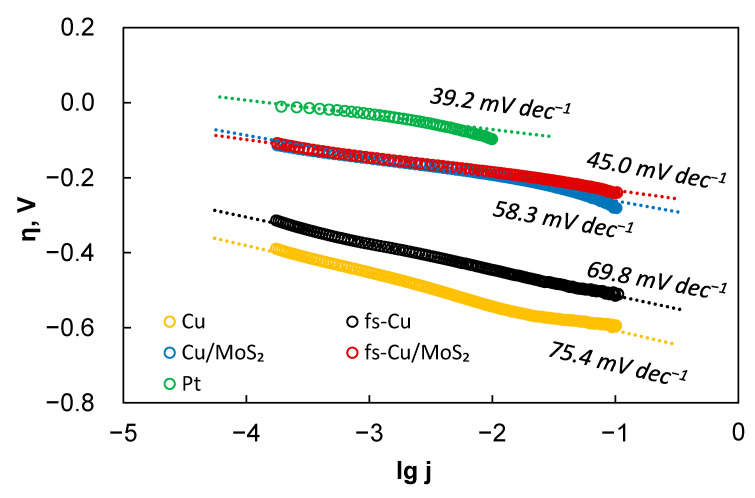
The Tafel slope calculations for the electrocatalytic electrodes prepared in this study. Data correspond to the LSV curves shown in Figure 3. Corrected for iR-drop.

Several important parameters that describe the activity of electrocatalytic films were extracted from these curves: the Tafel slope, exchange current density (*j*_0_), and the overpotential required to reach 10 mA cm^−2^ of HER current (η_10mA_). These data are presented in Table 2. Table 3 presents a broad comparison with other recent MoS_2_-based electrocatalysts for HER in acidic media. The Tafel slopes are calculated from the HER kinetic region where *lg j* is linearly related to the overpotential, as shown in Figure 4, as it is preferable to calculate the Tafel slopes from as large a current range as experimentally possible [39]. The Tafel slopes were calculated from a wide current density region from 0.1 mA cm^−2^ to ~50 mA cm^−2^, which allowed for a fairly accurate estimation. Certain Tafel slope values correspond to a particular mechanism of hydrogen evolution. The heterogeneously catalyzed hydrogen evolution reaction occurs through a combination of the Volmer hydrogen adsorption (Equation (2)), the Heyrovsky electrochemical recombination (Equation (3)), or the Tafel chemical recombination (Equation (4)) steps [40].

H_3_O^+^ + e^−^ + M ⇄ M-H + H_2_O (2)

M-H + H_3_O^+^ + e^−^ ⇄ H_2_ + H_2_O + M (3)

2M-H ⇄ H_2_ + 2M (4)

Hydrogen adsorption must always occur. A widely accepted rule of thumb is that if either the Volmer, Heyrovsky, or Tafel steps are rate-determining, then the observed Tafel slope will be 120, 40, or 30 mV dec^−1^, respectively [40,41]. Naturally, deviations and mixed kinetics do occur.

The results show that a slope of 75.4 mV dec^−1^ was observed on the Cu substrate. In comparison, the Tafel slope of fs-Cu (69.8 mV dec^−1^) suggests that the mechanism of HER on this electrode had not changed. Here, it must be mentioned that more recently, reasonable doubt has been cast onto the Tafel slope evaluation from potentiodynamic measurements, as they do not satisfy the true steady-state requirements unless carried out at extremely low scan rates [42]. Therefore, it is best to refrain from any mechanistic considerations from the Tafel slope values that are not standard. When an electrocatalytic MoS_2_ film is deposited on these substrates, the Tafel slopes typically observed for MoS_2_ [43] are as follows: 58.3 mV dec^−1^ for Cu/MoS_2_ and 45 mV dec^−1^ for fs-Cu/MoS_2_. This shows enhanced electrocatalytic activity and that hydrogen evolution occurs by the Volmer–Heyrovsky hydrogen adsorption/electrochemical recombination mechanism. Because the solution resistance is, in fact, not perfectly constant as the overpotential is increased, some over/undercorrection that manifests as a deviation from a linear trend can be seen at higher overpotentials.

**Figure 5:**
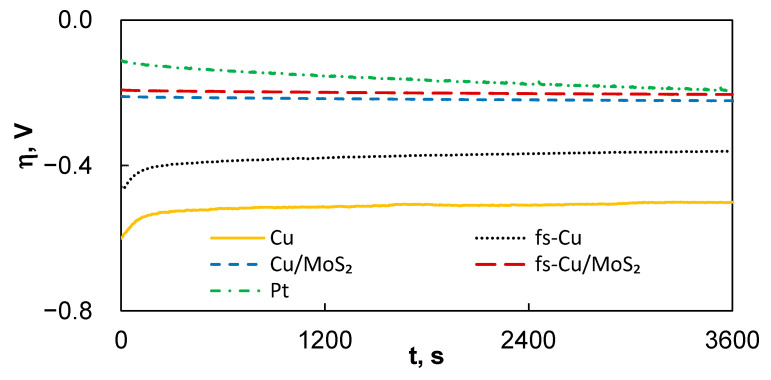
The overpotential-time curves that represent the electrochemical steady-state activity of the electrodes under 10 mA cm^−2^ of applied current density.

The electrochemical stability of an electrocatalytically performing electrode is also an important parameter, but there is no established standard estimation procedure in the scientific literature. For an industrial electrocatalyst device, two main parameters are of importance—on/off cycles and operational duration. In this study, the electrochemical stability of the electrodes was evaluated by applying a relatively mild constant current density of 10 mA cm^−2^ and registering the potential over the duration of 1 h, then by calculating how much their activity in terms of the initial η vs. end η had changed (Figure 5). Broadly speaking, over the course of the experiment for the uncoated substrates, the overpotential needed to maintain the set current decreased (Cu: −17%; fs-Cu: −21%), whereas for the electrodes with MoS_2_, it increased (Cu/MoS_2_: + 6%; fs-Cu/MoS_2_: +7%). That is, the Cu and fs-Cu gain some electrocatalytic activity, likely until the system approaches equilibrium. In contrast, the electrodes coated in an actively electrocatalytic MoS_2_ film lost activity over time, possibly due to the deactivation of the catalytically active sites by the reaction of surface sulfur with oxygen to form less active or even inert Mo-OH sites [47,48]. It is interesting to note that under these experimental conditions, plain Pt also lost a significant amount of catalytic activity, in fact, more than the Cu/MoS_2_ and fs-Cu/MoS_2_. Longer aging experiments would need to be conducted to investigate the industrial viability, but the electrodes are, at the very least, stable for more comprehensive electrochemical characterization.

### 3.3. Scanning Electrochemical Microscopy Study

As the fs-Cu/MoS_2_ electrodes had a uniform surface profile, it was useful to expand on their electrocatalytic activity characterization with a method that yielded a more local signal than the total electrode activity measurements. The SECM is a fairly new experimental technique that has recently been finding more use in the characterization of electrocatalytic macroelectrode surfaces [49,50,51]. In this study, a substrate-generation tip-collection mode was used, where the fs-Cu/MoS_2_ film was polarized in order to induce hydrogen evolution (generator), and the probe was set to oxidize the generated products (thus collector). The electrolyte here was the same as before—0.5 M H_2_SO_4_. The mechanism behind the processes occurring at the substrate and tip as well as between them are interesting and complex [51], but for the purpose of practical application, we assumed that H_2_ generates on the substrate (Equation (3)), the signal at the tip comes from the oxidation of H_2_ (Equation (5)), and that the H_2_ diffusion rate is steady at the tip measuring point.

H_2_ → 2H^+^ + 2e^−^ (5)

**Figure 6:**
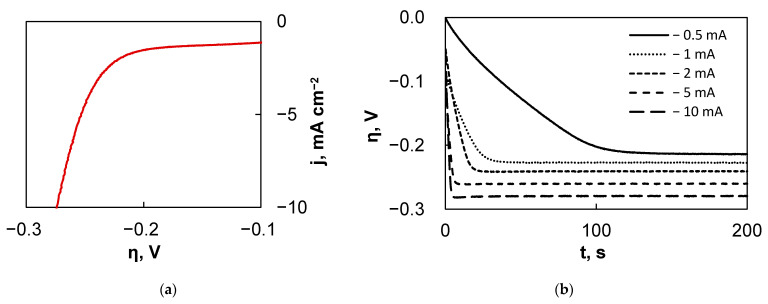

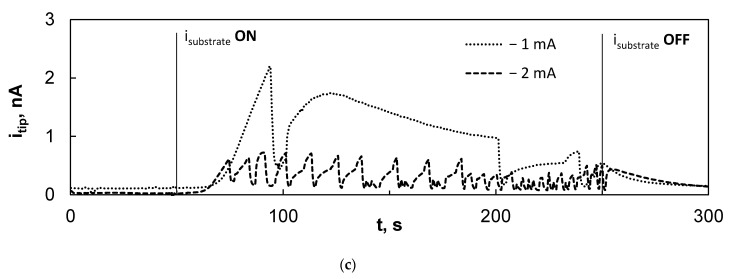
The results of the electrochemical behavior experiments of fs-Cu/MoS_2_ in the SECM: (**a**) LSV curve; (**b**) settling of the substrate overpotential under different applied currents; (**c**) the kinetics of the probe response to the substrate generation signal.

Several preliminary experiments were conducted in order to characterize the system and find the optimal conditions for mapping. First, after the fs-Cu/MoS_2_ sample was mounted into the SECM cell holder and the system was fully assembled, an LSV curve was obtained (Figure 6a), which provided information on the hydrogen bubbling location and its rate. Additionally, it was determined that galvanostatic conditions are preferential to apply in this mode, as this provides more reliable and reproducible results. Therefore, as a second experiment, the sample was set at certain current densities from −0.5 mA cm^−2^ to −10 mA cm^−2^ and the potential response was measured (Figure 6b). It is not surprising that larger applied currents correspond to a higher overpotential, but what is important is the time that it takes for the fs-Cu/MoS_2_ sample to approach the steady-state. Here, the larger the applied current, the sooner the sample reaches the steady-state conditions.

Finally, the electrochemical behavior of the Pt probe was examined. The 10 μm diameter probe was lowered to ~10 μm from the surface and polarized potentiostatically at 0 V vs. Ag/AgCl, because this was found to provide a sufficient signal to sample the HER in a previous experiment. For 50 s, the probe was kept at this potential and the sample at the OCP. Then, the current was applied to the sample for 200 s and again turned off at 250 s (Figure 6c). This experiment was important to understand the nature of the signal that can be expected. It was observed that the tip did register the signal from the sample, most likely through the oxidation of evolved H_2_ that is not gaseous, but dissolved in the solution. However, the tip current only began to increase at ~10 to 15 s after the current had been switched on. This is related to the time that it takes for the fs-Cu/MoS_2_ to reach the steady-state conditions, as shown in Figure 6b. The tip response time was faster when higher current densities were applied on the sample, but the *i_tip_* was lower (Figure S3). The tip current then rose sharply until a peak was reached, after which a severe drop occurred in most cases. This drop is likely to have been caused by the formation of gas bubbles on or around the probe tip, which blocks product diffusion and, consequently, the rate of the electrochemical reaction occurring at the tip. This presumed effect is particularly clear when −2 mA was applied on the sample and the probe response current appeared choppy. As the rate of the substrate HER increased (e.g., at −5 mA and −10 mA), the signal was heavily distorted by the mixing that is caused by the rapid evolution of gaseous hydrogen. The probe could even occasionally become blocked. However, it was assumed that in the SECM area scans, when the probe is mobile, this effect should be largely suppressed. Then, it is most important that the average *i_tip_* should be stable over the course of the scan.

**Figure 7:**
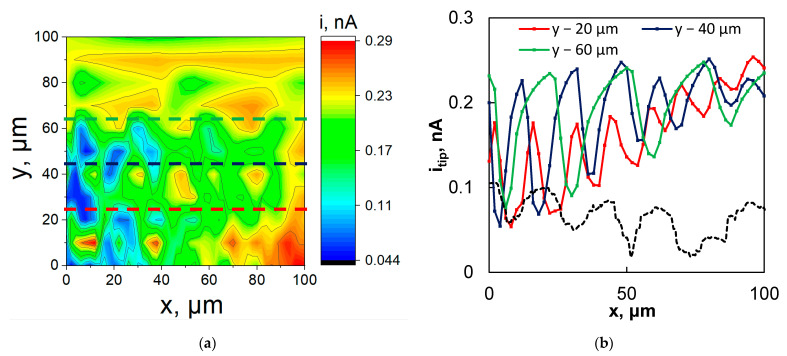
(**a**) The SECM map and (**b**) horizontal slices plus profilometry slice (from a different sample, only shown for reference) in the black dashed line of fs-Cu/MoS_2_. The map obtained in SG/TC mode. The sample galvanostatic at −2 mA, tip potentiostatic at 0 V vs. Ag/AgCl. Measured in 0.5 M H_2_SO_4_, probe scan rate 5 μm s^−1^, tip diameter 10 μm. X step size 2 μm, Y step size 10 μm.

Then, the XY area scans were obtained in order to observe the effect of the ridged morphology of the fs-Cu in the electrocatalytic activity of the fs-Cu/MoS_2_. Of course, any positive results should be attributed first and foremost to the topography of the surface. The SECM maps were obtained under several galvanostatic sample conditions (−1 mA, −2 mA, and −5 mA), but it was observed that −2 mA was the optimal condition that yielded the best signal. Although the 2D heatmap was not particularly revealing of the surface morphology (Figure 7a), slices across the *X* axis at several discrete Y values clearly reproduced the femtosecond-laser-induced ridges (Figure 7b). Errors in mapping could be attributed to the uneven sample mounting, but there is also no reason to expect that the distribution of the electrochemical HER activity on the sample is perfectly homogeneous. The SECM maps that were obtained at −1 mA and −5 mA are shown in Figure S4. When the substrate was set at −1 mA, not enough H_2_ was evolved to obtain a distinct localized electrocatalytic activity signal over most of the surface (hence, the flat 2D slices). Conversely, when the substrate was set at −5 mA, significant signal distortion occurred due to the vigorous evolution of gaseous H_2_.

The width of the fs-Cu peaks and pits as observed from the SECM mapping corresponded well to the results of the profilometric analysis. It must be noted that in this case, the SECM signal was caused by the oxidation of the substrate-generated products, so the map is a reflection of the radial diffusion of these products away from the sample. Because some of the 2D slices (e.g., Y − 40 μm and Y − 60 μm) reproduce the profilometric slice (Figure 7b, dashed line) so well, it may be inferred that the HER activity is similar in pits and on peaks. However, in some other cases such as Y − 80 μm to Y − 100 μm, the *i_tip_* was almost constant, as if scanning over a completely flat surface, as can be seen in Figure 7a. The SECM is a highly sensitive technique that is particularly difficult to apply when gas generation is occurring, but this case proved to be of moderate success and further confirmed that the laser-ablated morphology does have an impact on the performance of the electrode as an electrocatalyst.

### 3.4. Electrochemical Impedance Spectroscopy Study

Electrochemical impedance spectroscopy is a very useful tool in heterogeneous electrocatalyst characterization because it gives the ability to measure certain parameters that are related to the electrochemically active or even electrocatalytically active surface area of the electrode. In previous studies, EIS has been used to compare the electrocatalytic activities of electrodes with an uncertain geometrical surface area [34,52] and a similar approach was applied here. The EIS spectra were obtained at overpotentials where little to moderate hydrogen evolution occurs, depending on the catalytic activity of the electrode (−0.1 V, −0.2 V, −0.3 V).

**Figure 8:**
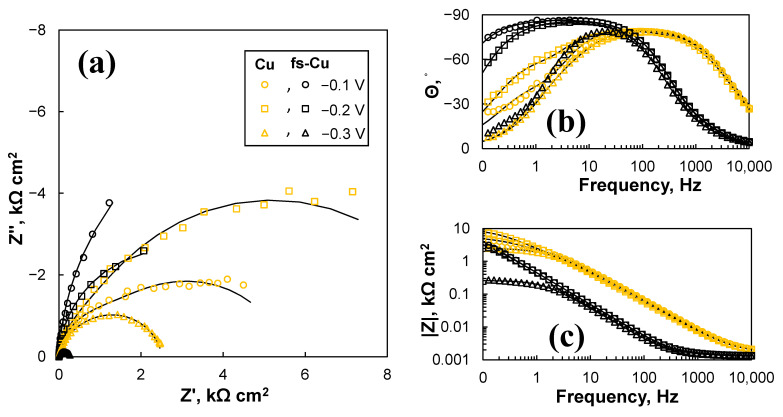
The EIS spectra of the plain Cu and fs-Cu surfaces in the (**a**) Nyquist complex coordinates and (**b**,**c**) Bode coordinates. Solid lines show equivalent circuit fits.

The EIS study, unsurprisingly, revealed that there was a significant difference in the electrocatalytic activity between the copper surfaces (Cu, fs-Cu) and those coated with catalytic films (Cu/MoS_2_, fs-Cu/MoS_2_). The spectra of the plain Cu surfaces are shown in Figure 8, and they were characterized by relatively large impedance magnitudes. For fs-Cu, at lower overpotentials, the impedance response was almost capacitive, as seen from the profile of the spectra in complex coordinates (Figure 8a) and the tendency of the phase shift *Θ* to approach −90° at low frequencies (Figure 8b). However, at −0.3 V, the impedance magnitude dropped substantially due to the onset of a Faradaic reaction, which must be HER under these conditions. The plain Cu exhibited similar tendencies, but its impedance magnitudes were larger. Judging from the phase shift behavior, the double layer charge/discharge kinetics were faster (because the phase maximum is reached at higher frequencies), but the appearance of a small hump at the intermediate frequencies (10 Hz to 1 Hz) suggests the onset of a Faradaic reaction. In comparison to fs-Cu, because Θ approached 0 again at 0.1 Hz, this effectively means that no significant low-frequency capacitive behavior (which could be related to hydrogen adsorption) was observed.

**Figure 9:**
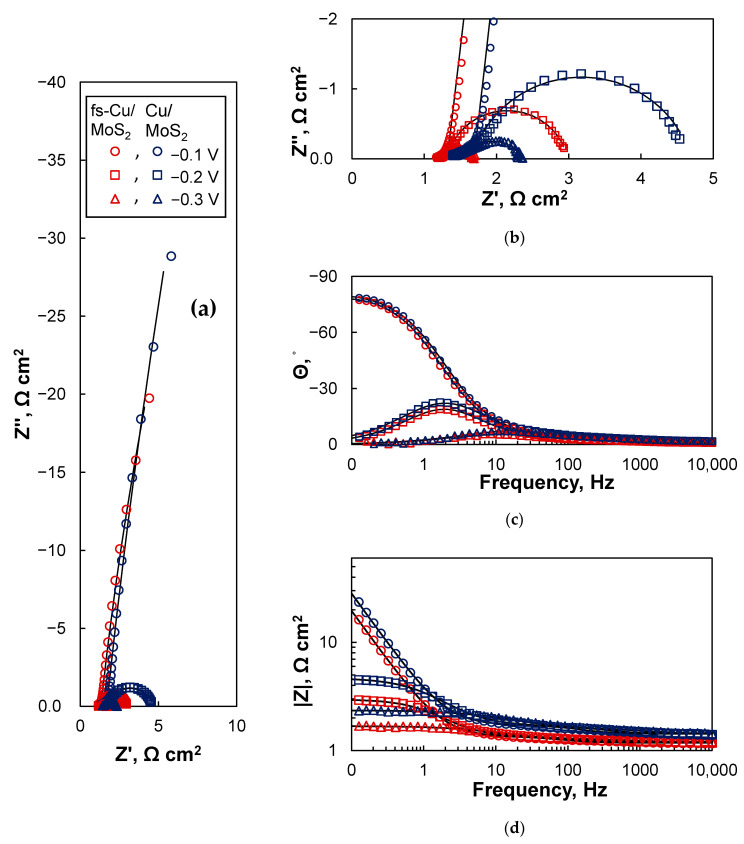
The EIS spectra of the Cu/MoS_2_ and fs-Cu/MoS_2_ electrodes in the (**a**,**b**) Nyquist complex coordinates and (**c**,**d**) bode coordinates. Solid lines were fit to the equivalent circuits shown in Figure 10.

The EIS spectra of the MoS_2_-coated electrodes were significantly different due to their superior electrocatalytic activity when compared to the uncoated copper surfaces (Figure 9). For both Cu/MoS_2_ and fs-Cu/MoS_2_ the spectra followed similar trends. A strongly capacitive response occurred at −0.1 V, as seen from the profile of the spectra in complex coordinates (Figure 9a,b) and the phase shift approaching −90° (Figure 9c). This is likely related to the adsorption of H^+^ to form a layer of H_ads_, because the Gibbs free energy of hydrogen adsorption on the sulfur vacancies/active sites of MoS_2−x_ is ΔG_H_^0^ ~−0.095 eV [53]. At higher overpotentials of −0.2 V and −0.3 V, hydrogen evolution began to occur at a rapid rate, and this is reflected in the spectra as the system turned from blocking to transmissive: semicircles were formed in complex coordinates (Figure 9b), Θ approached 0° at low frequencies (Figure 9c), and the impedance magnitude |Z| remained constant (Figure 9d). At first glance, there was little apparent difference between the impedances of the Cu/MoS_2_ and fs-Cu/MoS_2_ samples, but especially from Figure 9d, it can be inferred that the impedance magnitude of the fs-Cu/MoS_2_ was overall lower, which means that the system was more conductive/electrocatalytically active.

**Figure 10:**
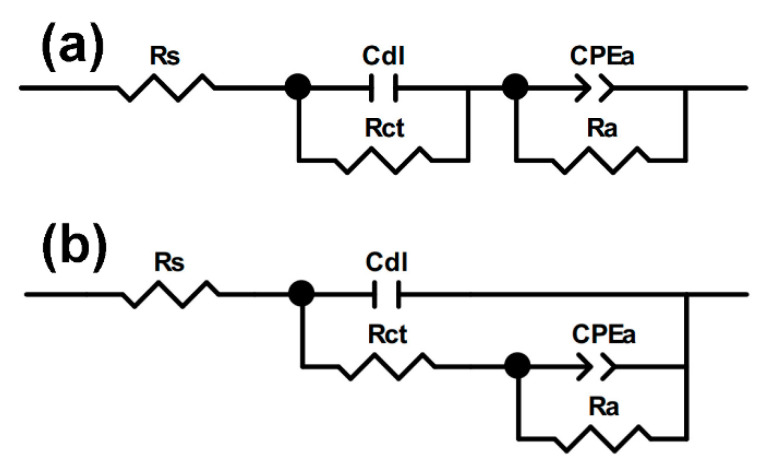
The equivalent electrical circuits that were used to fit the impedance spectra obtained in this study for (**a**) no/weak adsorption and (**b**) strong adsorption, where *R_s_* is the solution resistance, *C_dl_* is the double layer capacitance, *R_ct_* is the charge transfer resistance, and the constant phase element *CPE_a_* and *R_a_* are the adsorption-related capacitance and resistance, respectively.

The electrochemical impedance spectra were interpreted in terms of the equivalent electric circuits in order to discern and calculate the certain parameters that describe the surface/electrolyte interface. Two equivalent circuits were used for fitting: the uncoated Cu and fs-Cu (Figure 10a) and the electrodes on which a MoS_2_ film had been electrodeposited (Figure 10b). These circuits appeared similar, but they distinguished the different mechanisms of the electrode processes. The circuit shown in Figure 10a describes a system where two charge transfer processes can occur independently of each other, whereas in the second circuit, as shown in Figure 10b, the two processes must occur sequentially (i.e., the hydrogen adsorption followed by electrochemical recombination) [54].

In these equivalent circuits, the constant phase element (*CPE*) was used to account for the inhomogeneity in the surface coverage by adsorbed hydrogen. The *CPE* values were recalculated into the capacitance by Equations (6) and (7), colloquially known as Brug et al.’s effective capacitance equation [55,56].

(6) $$C_{a}=T_{a}^{\frac{1}{n}}{\left(\frac{1}{R_{s}}+\frac{1}{R_{a}}\right)}^{1-\frac{1}{n}}$$

(7) $$C_{a}=T_{a}^{\frac{1}{n}}{\left(\frac{1}{R_{s}+R_{ct}}+\frac{1}{R_{a}}\right)}^{1-\frac{1}{n}}$$

where *T_a_* are the values of the *CPE_a_* element.

The discrete parameters of the equivalent circuit correspond to certain measurable processes occurring at the catalyst/electrolyte interface. For example, the double layer capacitance *C_dl_* is measured at high perturbation frequencies and is related to the charge and discharge of the charge-compensating Helmholtz double layer. Therefore, it is proportional to the electrochemically active surface area of the electrode. This provides insights into the effective surface area of the electrodes. First of all, the *C_dl_* of fs-Cu is on average five times larger than that of unmodified Cu (Table 4). The *C_dl_* values for both of these electrodes remained relatively constant from −0.1 V to −0.3 V because this is a capacitive zone where no Faradaic reaction occurs. A different tendency was observed for the electrodes coated in a catalytic MoS_2_ film: *C_dl_* decreased with the overpotential (which is likely related to the increasing surface coverage of H_ads_). It is probably incorrect to compare the *C_dl_* values between the plain metal electrodes and MoS_2_ films because the mechanism and kinetics for the formation of a compensating double layer may be different. However, the *C_dl_* values of fs-Cu/MoS_2_ were ~3 times larger than of Cu/MoS_2_, indicating that the former had a larger electrochemically active surface area.

The second capacitance in the equivalent circuits (*CPE_a_*)—adsorption capacitance *C_a_*—is related to the adsorption/desorption of H^+^ on the electrode surface. The rate of this process is much slower than the charge/discharge of the Helmholtz double-layer, and is therefore observed at low perturbation frequencies. Because hydrogen evolution occurs via the adsorption (and subsequent electrochemical recombination) of H^+^ on the active site, this means that *C_a_* is proportional to the electrocatalytically active surface area. Here, it is most apparent that the *C_a_* of the Cu/MoS_2_ and fs-Cu/MoS_2_ samples (33–82 mF cm^−2^) was at least two magnitudes larger than the *C_a_* on the copper substrates (0.07–0.54 mF cm^−2^). This signifies that hydrogen adsorption occurred much more readily on the MoS_2_ material. As previously mentioned, the effect of the fs-ablated surface was apparent, as the *C_a_* of the fs-Cu/MoS_2_ was ~1.5 times larger than of Cu/MoS_2_.

It is interesting to note the observed peak for the Cu/MoS_2_ and fs-Cu/MoS_2_ *C_a_* values at −0.2 V. This appears to be in contrast to the expected result (that surface coverage by H_ads_ should increase with overpotential). This phenomenon can probably be attributed to the fact that *C_a_* (i.e., effective capacitance was recalculated from the dimensionless value of the constant phase element *CPE_a_*). Table S1 shows the values of *CPE_a_* and *n*. In the case of fs-Cu/MoS_2_, the *CPE_a_* values increased with the applied potential, but the *n* dropped from 0.92 at −0.1 V to 0.74 at −0.3 V. Here, *n* can be thought of as a coefficient that represents the inhomogeneity of the capacitive layer, and *n* decreases when the hydrogen evolution becomes more vigorous, and H_2_ gas bubbles disturb the catalyst/electrolyte interface.

The *R_ct_* and *R_a_* parameters are also worth discussing. Here, the charge transfer resistance *R_ct_* represents the resistance at the electrode/solution interface at high frequencies and is a measure of the system’s resistance to a unit of charge being transferred across the double layer. The values of *R_ct_* for fs-Cu, Cu/MoS_2_, and fs-Cu/MoS_2_ were from ~0.1 to 0.3 Ω cm^2^. The main difference of significance here is that the *R_ct_* of Cu was 1033 Ω cm^2^ at −0.1 V and dropped to 191 Ω cm^2^ at −0.3 V. As also suspected from the spectra in Figure 8a, this may signal the occurrence of a Faradaic reaction that is not hydrogen evolution.

**Figure 11:**
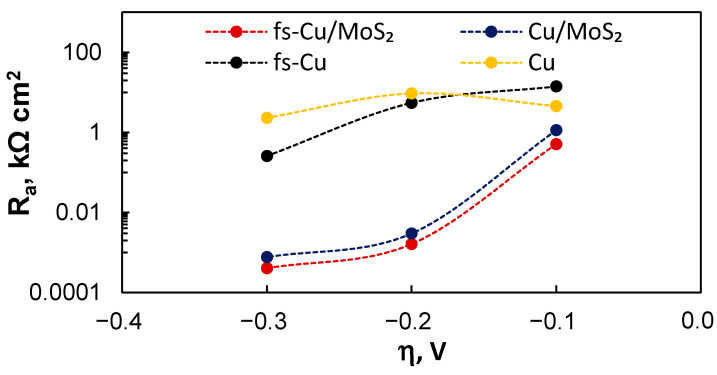
The equivalent circuit fitting results: the values of *R_a_* in relation to the overpotential.

*R_a_*, in particular, is representative of the electrode’s electrocatalytic HER performance. Mechanistically it is the resistance that H^+^ needs to overcome in order to adsorb on an active site and become H_ads_ (Equation (2)). Lower *R_a_* values correspond to faster HER kinetics and better electrocatalytic activity at a certain overpotential. The large difference between the copper substrates and MoS_2_ film was again evident (Figure 11). For Cu/MoS_2_ and fs-Cu/MoS_2_, the *R_a_* values were magnitudes smaller than those for their respective plain substrate electrodes. Moreover, the tendency of *R_a_* to decrease exponentially with the overpotential (corresponding to exponentially increasing the HER current as in Figure 3) was not observed for the substrate electrodes. When comparing Cu/MoS_2_ and fs-Cu/MoS_2_, the latter again exhibited measurably better electrocatalytic activity.

## 4. Conclusions

In this study, the femtosecond-laser-ablated copper surfaces were used as substrates with an elaborated surface area for the electrochemical deposition of MoS_2_ to create an efficient electrocatalytic cathode for hydrogen evolution in acidic media. Profilometric analysis was used to approximate the observable developed surface area of fs-Cu, which was ~40 times larger than the geometric surface area. However, after electrochemically depositing a MoS_2_ film onto this surface, the observed surface area decreased in half, which shows that extra care must be taken when approximating the surface area of heterogeneous catalysts with complex surface geometries. The fs-ablated (fs-Cu, fs-Cu/MoS_2_) and plain (Cu, Cu/MoS_2_) electrodes were characterized for their HER catalytic activity in 0.5 M H_2_SO_4_. It was observed that fs-Cu was more active than Cu, and similarly, fs-Cu/MoS_2_ surpassed Cu/MoS_2_, even though the same amount of catalyst had been deposited. Scanning electrochemical microscopy in a substrate generation/tip collection mode was used to examine the local HER activity of the fs-Cu/MoS_2_ films, and it was found that a signal could be obtained from both the pits and peaks, which suggests that hydrogen evolution occurs across the entire surface. Finally, electrochemical impedance spectroscopy was used to relate the surface structure/morphology to the electrocatalytic activity. The impedance spectra were fitted by equivalent electric circuits, and *C_dl_* and *C_a_* were calculated from the high- and low-frequency signals, respectively. The *C_dl_*, which is proportional to the electrochemically active surface area, was 2–5 times larger for fs-Cu and fs-Cu/MoS_2_ when compared to Cu and Cu/MoS_2_, which proves that the fs-ablated surfaces had a much larger electrochemically active surface area. Similarly, the *C_a_* of fs-Cu/MoS_2_ (which is proportional to the electrocatalytically active surface area) was larger than that of Cu/MoS_2_. This conclusively shows that using a laser-ablated surface for the electrochemical deposition of a catalyst yields a more efficient device that using planar substrates. These results show that femtosecond laser ablation, which is a scalable and controllable process, can be used to manufacture excellent substrates for heterogeneous electrocatalyst use.

## Figures and Tables

**Table 1:** The parameters calculated from the profilometric analysis.

	S_a,_ µm	S_v_, µm	S_p_, µm	S_par_, mm^2^	S_dar_, mm^2^
fs-Cu	6.86	53.11	24.26	0.25	10.46
fs-Cu/MoS_2_	4.94	48.36	16.21	0.25	4.68

**Table 2:** The values of the Tafel slopes, exchange current densities, and overpotential needed to reach 10 mA cm^−2^ of the HER current of the samples considered in this study.

Sample	Tafel Slope, mV dec^−1^	j_0_, µA cm^−2^	η_10mA_, mV
Cu	75.4	0.00089	−552
fs-Cu	69.8	0.0043	−445
Cu/MoS_2_	58.3	3.23	−193
fs-Cu/MoS_2_	45.0	0.65	−185
Pt	39.2	150	−97

**Table 3:** The comparison of the reported Tafel slopes and overpotentials at the 10 mA cm^−2^ HER current values (in 0.5 M H_2_SO_4_) of the MoS_2_-based electrocatalysts.

Sample	Synthesis Method	Tafel Slope, mV dec^−1^	η_10mA_, mV	Reference
rGO/MoS_x_−100 °C	Hydrothermal	48.8	−125	[27]
MoS_x_	Electrodeposition	55	−211	[28]
67% S_br_-MoS_x_	Chemical synthesis	46	−96	[29]
MoS_6_	Anodic electrodeposition	-	−161	[33]
Cu-foam/MoS_2_	Electrodeposition	49	−142	[34]
MoS_x_-TiO_2_NT	Electrodeposition	43	−73	[44]
Co-doped MoS_2_	In situ sulfuration	-	−155	[45]
Pd_0.2_-MoS_2_	Solvothermal	60	−106	[46]
Cu/MoS_2_	Electrodeposition	58.3	−193	This work
fs-Cu/MoS_2_	Electrodeposition	45.0	−185	This work

**Table 4:** The equivalent circuit fitting results and fitting errors.

**Cu**				
η, V	*C_dl_*, mF cm^−2^	*R_ct_*, Ω cm^2^	*C_a_*, mF cm^−2^	*R_a_*, kΩ cm^2^
−0.1	0.0529 ± 0.0035	1033 ± 32	0.176 ± 0.0091	4.53 ± 0.049
−0.2	0.114 ± 0.0089	546 ± 21	0.113 ± 0.0022	9.41 ± 0.069
−0.3	0.126 ± 0.018	191 ± 13	0.072 ± 0.0021	2.32 ± 0.011
**fs-Cu**				
−0.1	0.607 ± 0.063	0.164 ± 0.0096	0.387 ± 0.0013	14.1 ± 0.33
−0.2	0.582 ± 0.063	0.169 ± 0.010	0.390 ± 0.0015	5.49 ± 0.063
−0.3	0.644 ± 0.23	0.138 ± 0.027	0.544 ± 0.010	0.258 ± 0.0028
**Cu/MoS_2_**				
−0.1	3.76 ± 0.33	0.320 ± 0.013	49.6 ± 0.53	1.14 ± 0.068
−0.2	2.03 ± 0.19	0.264 ± 0.012	58.9 ± 1.7	0.00299 ± 4.3 × 10^−5^
−0.3	0.805 ± 0.073	0.149 ± 0.0083	33.6 ± 1.8	0.000768 ± 1.3 × 10^−5^
**fs-Cu/MoS_2_**				
−0.1	10.2 ± 0.90	0.206 ± 0.011	64.9 ± 0.97	0.510 ± 0.16
−0.2	6.69 ± 0.51	0.155 ± 0.0069	82.7 ± 1.8	0.00163 ± 1.7 × 10^−5^
−0.3	2.64 ± 0.49	0.073 ± 0.0082	63.3 ± 5.6	0.000405 ± 1.3 × 10^−5^

## Data Availability

The data presented in this study are available on request from the corresponding author.

## Supplementary Materials

The following supporting information can be downloaded at: https://www.mdpi.com/article/10.3390/ma15113926/s1, Figure S1: Stitched 3D and 2D profilograms of the fs-Cu and fs-Cu/MoS_2_ at 500 × 500 μm dimensions; Figure S2: The XRD patterns of the plain Cu, fs-Cu, and fs-Cu/MoS_2_, Figure S3: The *i_tip_* response to the substrate signal at different applied currents on the fs-Cu/MoS_2_ substrate; Figure S4: The SECM area maps and 2D slices of an fs-Cu/MoS_2_ film when the substrate was galvanostatically set at: (a,b)—1 mA; (c,d)—5 mA; Table S1: The T_a_ and n values of the *CPE_a_* element (in reference to Equation (7)).
