# Supplementary material for: Femtosecond Laser-Ablated Copper Surface as a Substrate for a MoS2-Based Hydrogen Evolution Reaction Electrocatalyst
Source: Materials (Basel). 2022 May 31;15(11):3926. doi: 10.3390/ma15113926 (PMC9182345; doi:10.3390/ma15113926)
Supplement: Supplementary file 1 [file materials-15-03926-s001.zip › materials-1724108-supplementary.pdf]

Supplementary material

Femtosecond laser-ablated copper surface as a substrate for a MoS_2-based hydrogen evolution reaction electrocatalyst

Ramūnas Levinas [1, 2, *], Asta Grigucevičienė [1], Tadas Kubilius [3], Aidas Matijošius [4], Loreta Tamašiūnaitė-Tamašauskaitė [1], Henrikas Cesiulis[2], and Eugenijus Norkus [1, *]

[1] State Research Institute, Center for Physical Sciences and Technology (FTMC), Saulėtekio Ave. 3, LT-10257 Vilnius, Lithuania; asta.griguceviciene@ftmc.lt (A. G.); loreta.tamasauskaite@ftmc.lt (L. T.); eugenijus.norkus@ftmc.lt (E. N.)

[2] Faculty of Chemistry and Geosciences, Vilnius University, Naugarduko str. 24, LT-03225 Vilnius, Lithuania; henrikas.cesiulis@chf.vu.lt (H. C.);

[3] Hydrogen Solutions Ltd., Partizanų str. 61-806, Kaunas LT-49282, Lithuania; tk@h2solutions.lt (T. K.)

[4] Laser Research Center, Vilnius University, Saulėtekio Ave. 10, Vilnius LT-10223, Lithuania; aidas.matijosius@ff.vu.lt (A. M.)

* Correspondence: ramunas.levinas@ftmc.lt (R. L.); eugenijus.norkus@ftmc.lt (E. N.)

Table of contents

Figure S1. Stitched 3D and 2D profilograms of fs-Cu and fs-Cu/MoS_2 in 500 x 500 µm dimensions.

Figure S2. XRD patters of plain Cu, fs-Cu, and fs-Cu/MoS_2.

Figure S3. i_{tip} response to substrate signal at different applied currents on the fs-Cu/MoS_2 substrate.

Figure S4. SECM area maps and 2D slices of an fs-Cu/MoS_2 film when the substrate is galvanostatically set at: (**a, b**) – 1 mA; (**c, d**) - 5 mA.

Table S1. T_a and n Values of the CPE_a element (in reference to equation 7).

Section S3: Results

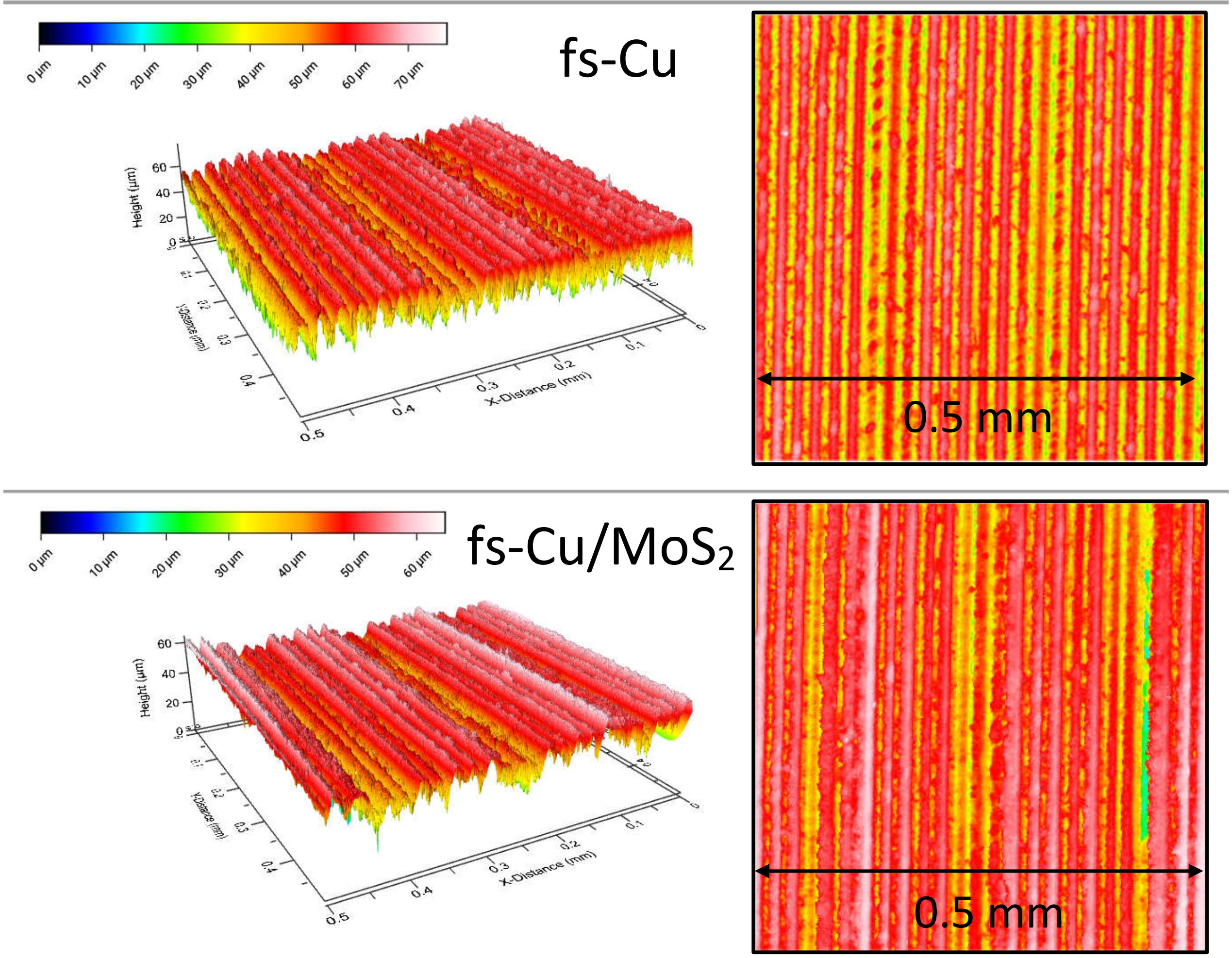

Figure S1. *Stitched 3D and 2D profilograms of fs-Cu and fs-Cu/MoS_2 in 500 x 500 µm dimensions.*

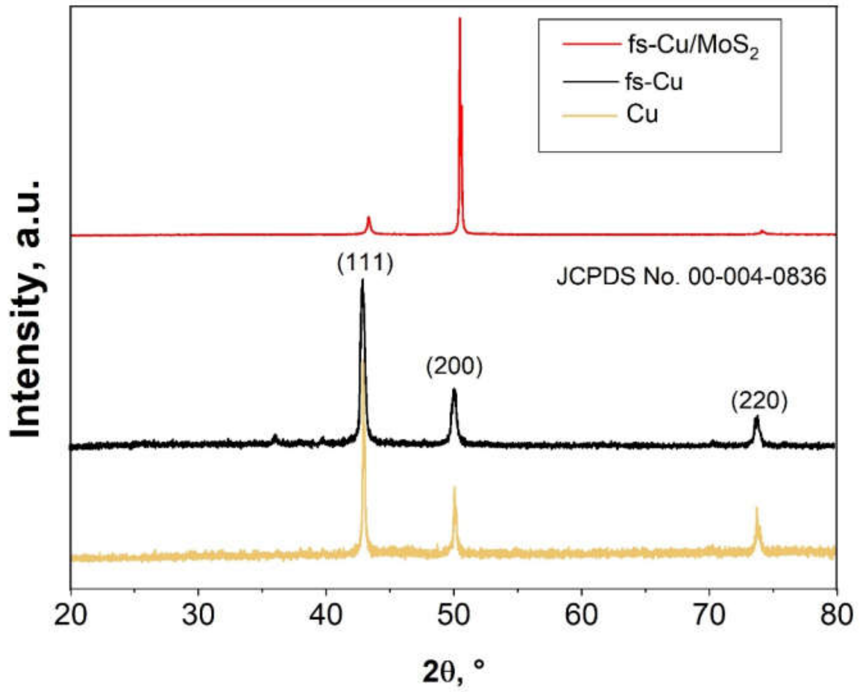

Figure S2. *XRD patters of plain Cu, fs-Cu, and fs-Cu/MoS_2.*

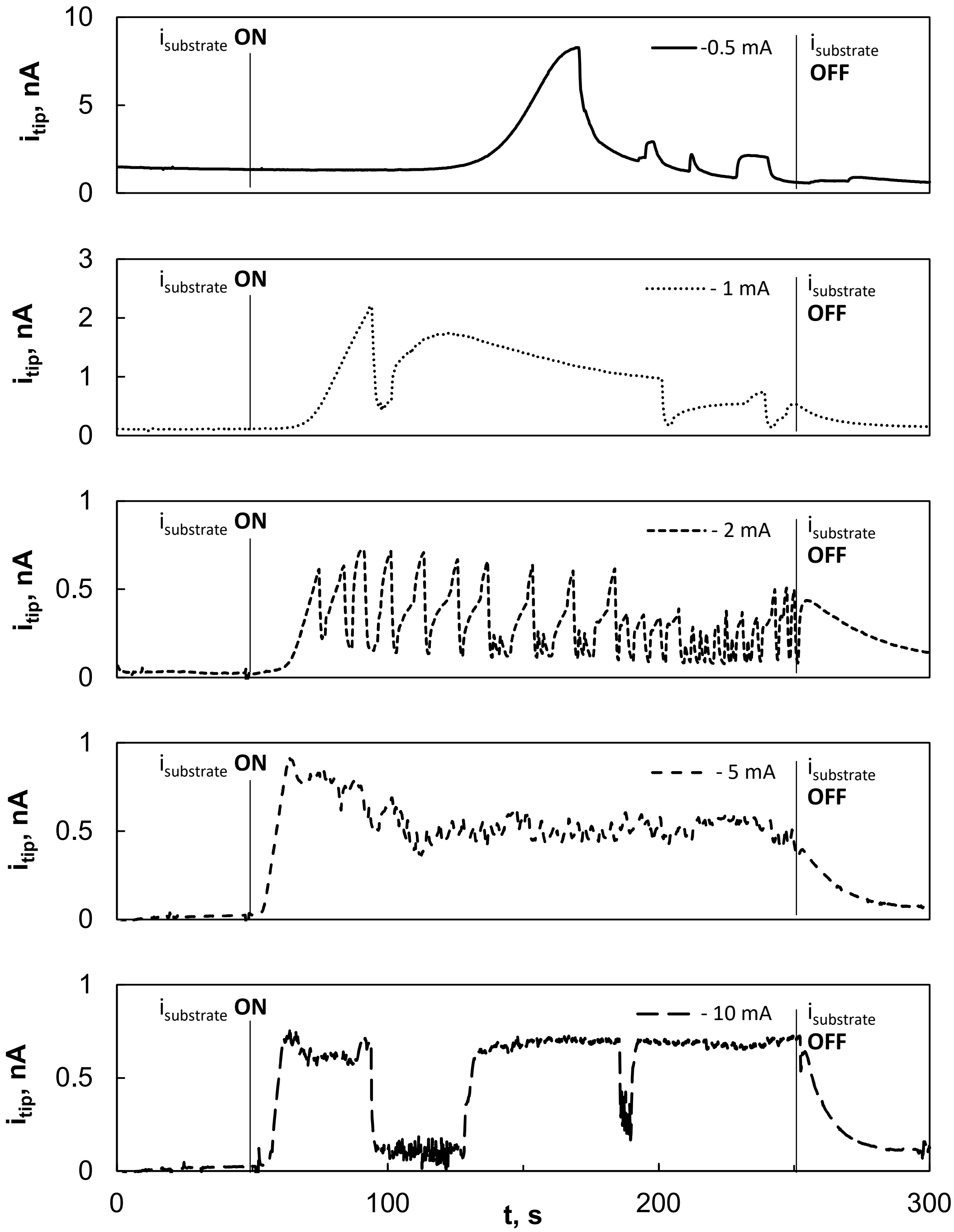

Figure S3. *i_{tip} response to substrate signal at different applied currents on the fs-Cu/MoS_2 substrate.*

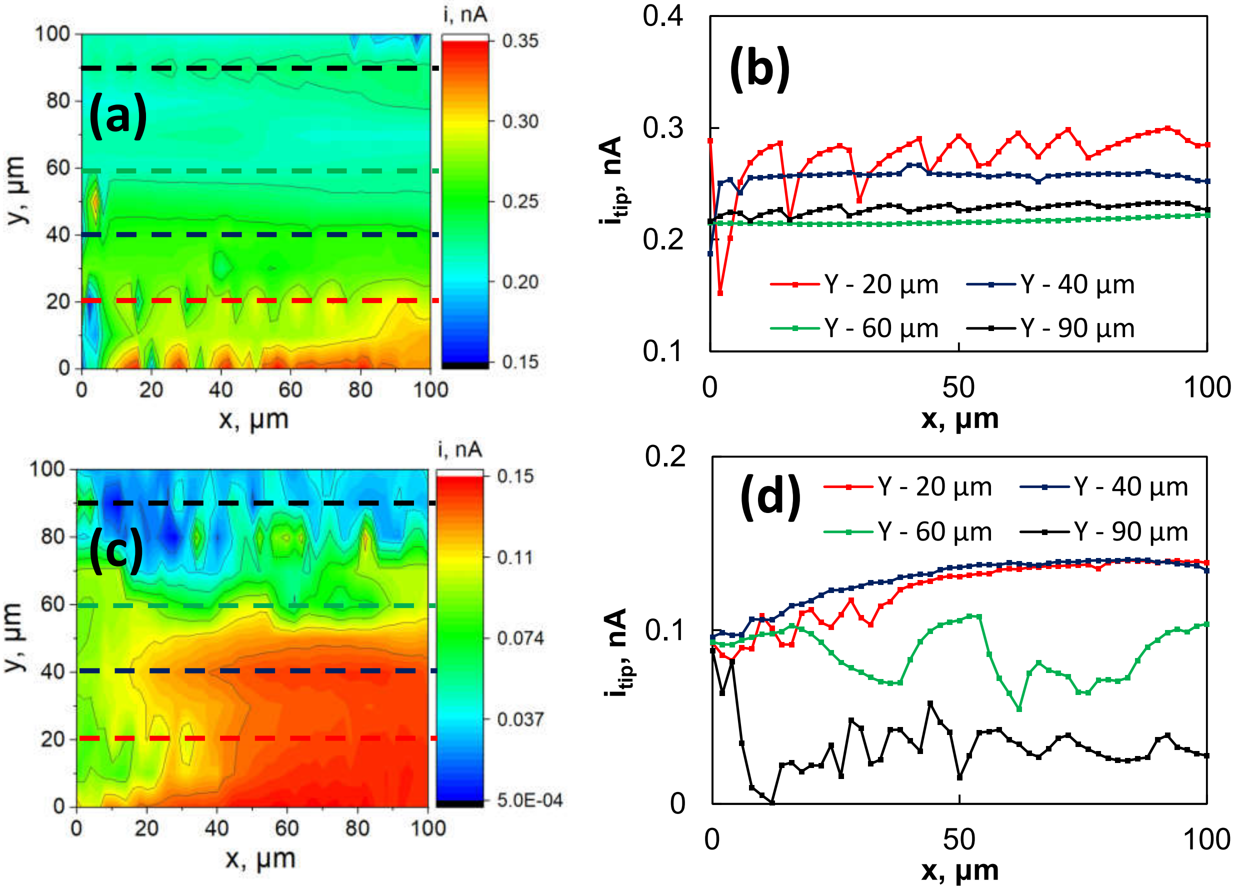

Figure S4. *SECM area maps and 2D slices of a fs-Cu/MoS_2 film when the substrate is galvanostatically set at: **(a, b)** - 1 mA; **(c, d)** - 5 mA.*

Table S1. T_a and *n* Values of the CPE_a element (in reference to equation 7).

Cu			
η, V	T_a of $CPE_a \cdot 10^{-3}$	n	C_a, mF cm^{-2}
-0.1	0.133 ±0.014	0.833 ±0.0048	0.176 ±0.0091
-0.2	0.0902 ±0.0036	0.863 ±0.0020	0.113 ±0.0022
-0.3	0.0610 ±0.0036	0.895 ±0.0022	0.0721 ±0.0021
**fs-Cu**			
-0.1	0.372 ±0.0012	0.969 ±0.00078	0.387 ±0.0013
-0.2	0.374 ±0.0014	0.968 ±0.00087	0.390 ±0.0015
-0.3	0.514 ±0.0096	0.955 ±0.0037	0.544 ±0.010
**Cu/MoS_2**			
-0.1	51.1 ±0.55	0.93 ±0.0047	49.6 ±0.53
-0.2	67.3 ±1.9	0.83 ±0.011	58.9 ±1.7
-0.3	59.9 ±3.2	0.70 ±0.014	33.6 ±1.8
**fs-Cu/MoS_2**			
-0.1	69.2 ±1.0	0.922 ±0.0040	64.9 ±0.97
-0.2	97.9 ±2.1	0.870 ±0.0084	82.7 ±1.8
-0.3	128 ±11	0.736 ±0.025	63.3 ±5.6
